# A Study on Determining Time-Of-Flight Difference of Overlapping Ultrasonic Signal: Wave-Transform Network

**DOI:** 10.3390/s20185140

**Published:** 2020-09-09

**Authors:** Zhipeng Li, Tong Wu, Wei Zhang, Xuyang Gao, Zhenqiu Yao, Yanjun Li, Yibing Shi

**Affiliations:** 1 School of Automation Engineering, University of Electronic Science and Technology of China, Chengdu 611731, China; lizhipeng1202@std.uestc.edu.cn (Z.L.); 201822060431@std.uestc.edu.cn (T.W.); xuyanggao@std.uestc.edu.cn (X.G.); yjli@uestc.edu.cn (Y.L.); ybshi@uestc.edu.cn (Y.S.); 2 Glasgow College, University of Electronic Science and Technology of China, Chengdu 611731, China; 2017200504010@std.uestc.edu.cn

**Keywords:** thickness measurement, ultrasonic signal, TOFD, deep learning

## Abstract

An ultrasonic sensors system is commonly used to measure the wall thickness of buried pipelines in the transportation of oil and gas. The key of the system is to precisely measure time-of-flight difference (TOFD) produced by the reflection of ultrasonic on the inner and outer surfaces of the pipelines. In this paper, based on deep learning, a novel method termed Wave-Transform Network is proposed to tackle the issues. The network consists of two parts: part 1 is designed to separate the potential overlapping ultrasonic echo signals generated from two surfaces, and part 2 is utilized to divide the sample points of each signal into two types corresponding to before and after the arrival time of ultrasonic echo, which can determine the time-of-flight (TOF) of each signal and calculate the thickness of pipelines. Numerical simulation and actual experiments are carried out, and the results show satisfactory performances.

## 1. Introduction

**Figure 1:**
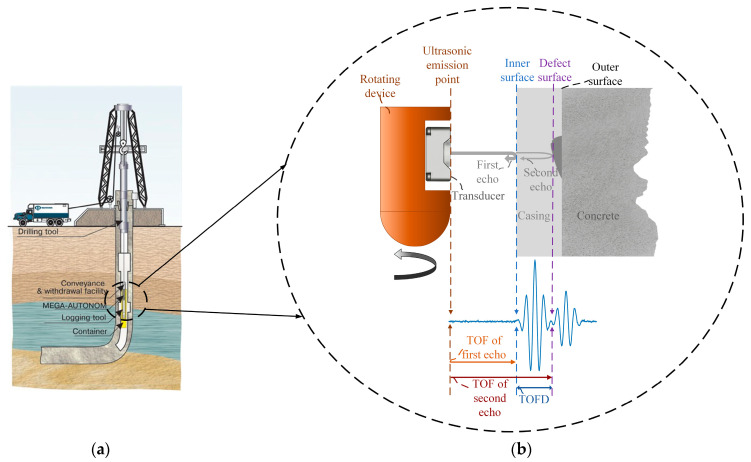
Overview of ultrasonic non-destructive testing in buried pipelines. (**a**) The structure of drilling well; (**b**) The principle of thickness measurement of buried pipelines via ultrasonic.

At low density and high strength, well cementation and completion can be prepared for reservoir reconstruction and later production, which is a critical technology in petroleum exploration. The structure of well is shown as Figure 1a [1], and the steel pipe (called casing) is in the innermost layer of the well where the casing is placed to seal the complex stratum which is easy to collapse and leakage, to consolidate the drilled hole, and to ensure propitious drilling. Hence, pipeline integrity is of crucial importance to engineers, which can ensure the safety of operators, productivity and environmental compliance. Compared with eddy current testing [2], radiographic testing [3], and data-driven approach [4], the ultrasonic non-destructive testing (NDT) [5] can detect potential internal defects of pipes with low cost and invert thickness of pipelines accurately. For this reason, ultrasonic pulse-echo approaches have been utilized extensively in the analysis of thickness and bond quality of pipelines [6]. For instance, a cement-based drinking water pipeline is monitored inline by the ultrasonic pulse echo method [7]. However, because of the pipelines being buried deeply, it is impossible to dig out the pipes for thickness detection, which means that the transducers are only placed inside the pipeline and detect the potential cracks. Hence, an instrument shown in Figure 1a is manufactured to measure the thickness of casing; the time that ultrasonic wave passes from transmitting to receiving is called time-of-flight (TOF). Figure 1b illustrates this measurement principle that the thickness can be calculated by the time-of-flight difference (TOFD) of ultrasonic between the inner and outer wall of the casing, as shown in Equation (1).

(1) $$D_{th}=V_{m}\times \frac{t_{TOFD}}{2}=V_{m}\times \frac{t_{TOF2}-t_{TOF1}}{2},$$

where $V_{m}$ is the velocity of ultrasonic in buried pipelines; $t_{TOF1}$ is a TOF of the first ultrasonic echo reflecting from the inner surface of the casing; $t_{TOF2}$ is a TOF of the second ultrasonic echo reflecting from the outer surface of the casing; $D_{th}$ is the thickness of the casing. The thickness of the entire pipeline in the drilling well can be measured by moving the instrument from up to down and rotating the fixed transducer. The key of thickness measurement is to determine the TOFD, that is, determine the TOF1 and TOF2 precisely. 

**Figure 2:**
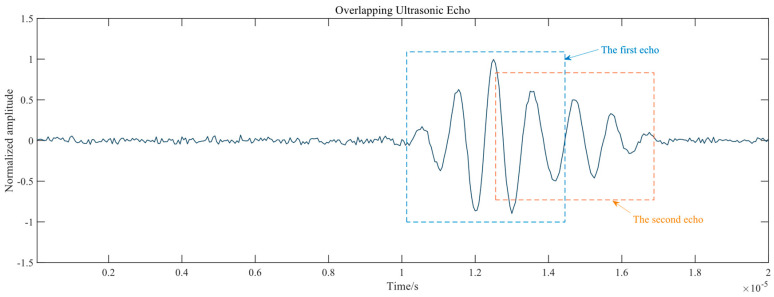
Overlapping ultrasonic echo acquired where the thickness of pipeline is too thin.

When working time of pipelines increase, metal loss caused by erosion is a widespread issue in the oil and gas (O&G) and Power Generation industries [8], and it can result in defects and decreasing thickness. For this reason, the transducer may receive overlapping echo signals, as shown in Figure 2. Therefore, before determining the TOF of each echo signal, the separation of overlapping ultrasonic echo must be performed in the first stage [9]. There have been considerable methods concerning single-channel blind source separation (SCBSS) utilized to cope with this serious issue, including matrix transformation; filtering in transform domain; sparse decomposition representation, etc. On the basis of transform domain, D M J Cowell and S Freear assume the ultrasonic echo signal as a linear frequency modulated (LFM) signal, and transform the signal from the time domain to the fractional domain. Each component of overlapping echo signal can be gained by filtering other components using window functions. Via the Inverse Fractional Fourier Transform (IFrFT) of every separated component in the fractional domain, each single component of echo signal can be obtained [10]. Zhenkun Lu et al. further discuss how to determine the optimal transform order of FrFT for the decomposition of overlapping ultrasonic echoes [11,12]. This kind of method can only work when the target mixed ultrasonic signal is assumed as the LFM signal, which has the obvious separated feature in the fractional domain. For the separation of multi-channel signals, independent component analysis (ICA) based on matrix transformation is the primary method for multi-channel overlapping signals. Shanshan Hou et al. adopt this approach and realize the transformer partial discharge (PD) direct wave ultrasonic signal separation [13]. However, SCBSS is an ill-conditioned problem, lacking sufficient information to obtain a unique solution; that is, the method is only appropriate for multi-channel signals. Sparse decomposition representation is derived from the research on the principle of biological auditory nerve processing information by Smith [14]. Etai Mor et al. propose a sparse approximation method for overlapping ultrasonic echoes, which modifies the matching pursuit (MP) method to achieve approximations in which each signal echo is approximated by a single atom function possessing a plain physical interpretation [15]. Depending upon the completeness of atoms, the method could not separate potential mixed signal if the atoms are unable to cover all features of actual sample signals. Furthermore, noise has an adverse impact on the result of separation. The second stage is to determine the TOF of each separated echo signal. In terms of the single ultrasonic echo signal, research on TOF has been carried out by researchers around the world who have created plenty of methods to tackle the issue. All existing methods can be roughly divided into two categories corresponding to the mathematical model including model-based estimation of ultrasonic echoes [16]; Kalman filtering for TOF Estimation [17]; echo signal envelope [18], etc., and to energy distribution including peak-value; information criteria [19,20]; higher-order statistics method [21]; Teager–Kaiser energy operator (TKEO) [22], etc.

With the recent surge in developments of deep learning, audio signal processing has adopted this method to extensively tackle problems that were difficult to solve in the past, such as source separation, audio enhancement, and generative models for speech, sound, and music synthesis [23]. Aiming at single-channel audio source separation, Felix Weninger et al. conceived a method based on Recurrent Neural Networks to obtain clean speech signals from single-channel recordings with non-stationary noises [24]. Emad M. Grais et al. presented a Deep Neural Network (DNN) using superior models trained by highly variable real-world signals and then used an energy minimization objective to estimate the sources and their gains during the separation stage [25]. Furthermore, they proposed a two-stage DNN approach, which is used to map the features of the mixed-signal into the features of the sources directly; the second part is that the separated sources are enhanced using DNN to decrease the interference between the separated sources and distortions [26].

In this paper, we design a new neural network termed Wave-Transform Network (WT-net) containing two parts to accomplish the end-to-end computation of TOFD: part 1 is the separation network separating overlapping ultrasonic echo; while achieving the separated single echo, part 2 is used to determine the TOF of each echo signal. In the following paper, Section 2 demonstrates the detailed architecture of WT-net; Section 3 presents the performance of the proposed network compared with other methods, and performs the actual experiments; and Section 4 summarizes the entire method and proposes further work.

## 2. Methods

### 2.1. Overview of WT-Net

**Figure 3:**
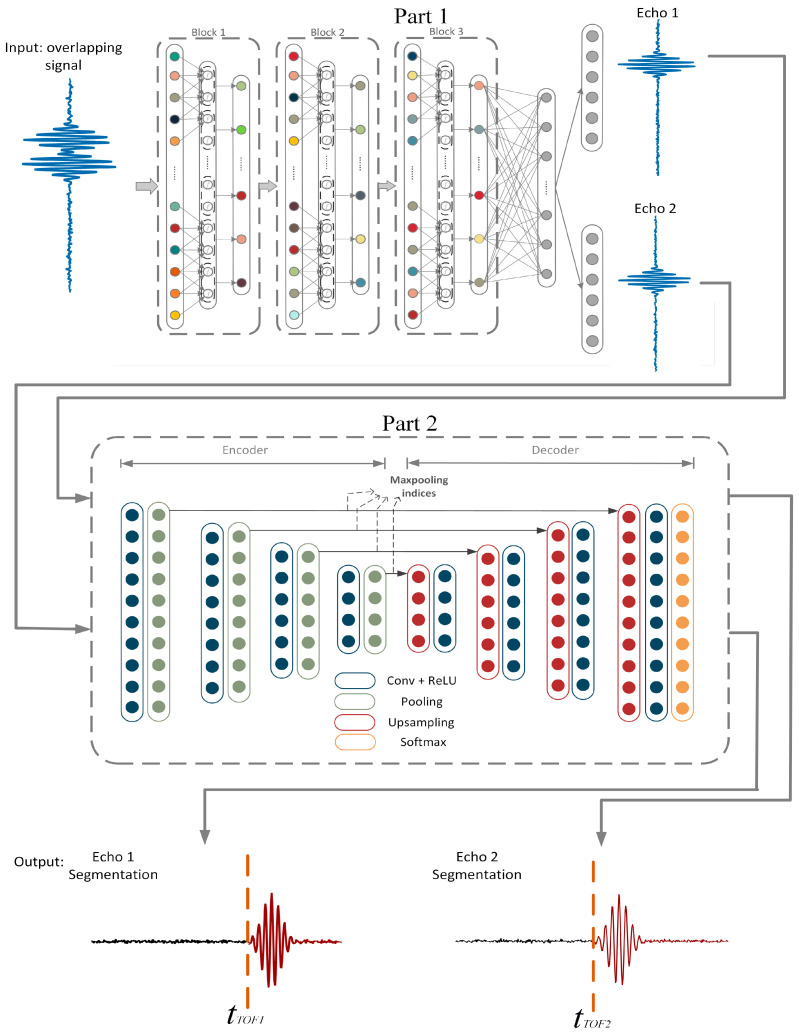
An illustration of the WT-net architecture including Part 1, S-net, and Part 2, D-net. S-net is based on 1D-CNN, which utilizes trainable filter kernels to extract the potential features in overlapping echo signals and achieve the single component. D-net further determines the TOF of each independent signal via semantic sample points segmentation. All sample echo points are divided into two categories corresponding to before and after TOF. The boundary point between two categories denotes the TOF.

As mentioned in the introduction, the proposed model, WT-net, includes part 1, separation net (S-net), and part 2, determination net (D-net), corresponding to the overlapping echo separation and TOF measurement of each signal, respectively. Two independent parts of the net are required to be trained, respectively. S-net is designed to separate the ultrasonic echo signals. As distinct from DNN in [25], the base-net of part 1 is one-dimensional convolution neural network (1D-CNN) which is set to capture the local feature better. The output of S-net is a single component of overlapping ultrasonic echo, and it is utilized as the input of D-net. D-net is a 1D fully convolutional neural network architecture for semantic sample points segmentation. All sample points of echo signal are divided into two categories corresponding to before and after the TOF, and the boundary point between two categories denotes the TOF of each separated echo signal. The architecture of the WT-net is illustrated in Figure 3.

### 2.2. Part 1: S-Net

The ultrasonic echo signal reflected from casing may overlap because of the too thin thickness of the wall in pipelines. Accordingly, the first stage of the algorithm is to decompose the overlapping signal into the single constituents, and S-net aims to accomplish this target. Inspired by [15] and [25], in this paper, we propose a method based on the learning approach to separate the target signals. Differing from dictionary learning and the generic DNN, the proposed network is based on 1D-CNN that uses various 1D convolution filter kernels to match different features in original overlapping signals, which are able to find the relationships between original overlapping signals and every single component in the overlapping signals. The entire structure of S-net is demonstrated in Table 1. 

The mean-squared-error (MSE) is selected as loss function in S-net training. Hence, the optimized objective is given in the following equation

(2) $$E_{mse}=\frac{1}{n}\sum_{i=1}^{n}{\left(y_{N_{i}}-y_{n_{i}}\right)}^{2}$$

where $y_{N_{i}}$ denotes true label sample point of the target echo signal, $y_{n_{i}}$ denotes the predicted output of S-net and *n* denotes the number of sample points of each ultrasonic echo in which there are 800 points because we splice two 400 × 1 output single echo signal into one 800 × 1 signal.

### 2.3. Part 2: D-Net

As mentioned in the introduction, existing methods measuring the TOF of ultrasonic echo is based on the mathematical model or energy distribution. In this section, we try to tackle the issue from a different perspective to enhance the estimation performance of TOF.

In a digital signal processing (DSP) system, all signals are sampled as discrete points with different sampling frequencies. Each sample point indicates a situation at a particular time during the sampling process [27]. In this paper, we define all sample points received by the transducer as being divided into two categories corresponding to before and after the TOF of ultrasonic echo. Hence, the objective to determine the TOF is changed into finding an optimal segmentation between two categories in sample points. 

Inspired by semantic segmentation in computer vision [28,29], we design the D-net, which is an Encoder-Decoder architecture network to accomplish the sample points-level classification. This framework is illustrated in the Part 2 of Figure 3. D-net is divided into two subnets: an encoder network and a corresponding decoder network which is connected with an ultimate sampled-pointwise classification layer. The encoder network comprises ten convolutional layers for object classification. Each encoder layer has a corresponding decoder layer, and accordingly, the decoder network has ten layers. The ultimate decoder output is sent to a multi-category softmax classifier to generate class probabilities for each sample point independently. 

**Figure 4:**
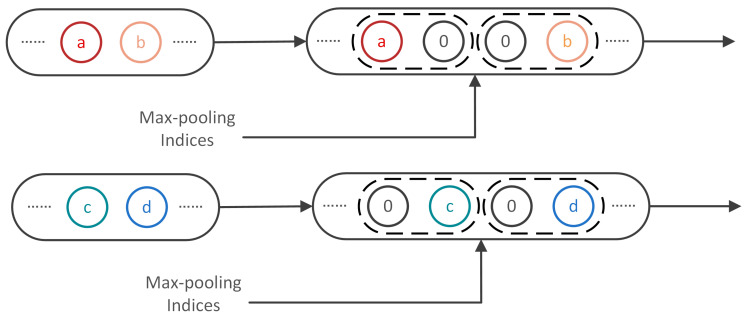
Max-pooling indices technique. a, b, c, d correspond to values in a feature map. D-net adopts the max-pooling indices storing the location information in max-pooling layer to upsample the feature map which further is sent to convolve with a trainable decoder filter bank.

Each encoder in the encoder network implements convolution with different convolution kernels to produce a set of feature maps. Then ReLU, which is used as an activation function, is to obtain a non-linear output of the convolution result. Following that, max-pooling with a window 1 × 2 and stride 2 is used to actualize the translation invariance over small time-domain shifts in the input signals. To achieve the location information of sample points in the decoding stage, it is essential to capture and store the boundary location of different categories of sample points in the encoder feature maps before sub-sampling is performed. Hence, max-pooling indices, which are the locations of the maximum feature value in each pooling window, are utilized to store the location of the sub-sample point sampled from the signal at the upper layer. The decoder network upsamples its input feature maps with the stored max-pooling indices from the corresponding encoder feature maps, which can produce sparse feature maps. Figure 4 illustrates the technique of max-pooling indices.

Followed by the convolution layers with a trainable decoder filter bank, these sparse feature maps can produce dense feature maps. Finally, the feature maps of the last convolution layer are sent to a trainable softmax classifier. This softmax distinguishes each category of all sample points into two classes corresponding to before and after the TOF of echo, independently. The predicted segmentation corresponds to the category with maximum probability at each sample point. The specific structure of D-net is shown in Table 2. 

### 2.4. Dataset

S-net and D-net work independently in the proposed method. Hence, we train and validate them in their respective datasets. In order to train the S-net, simulated ultrasonic echo model, which simulates ultrasonic echo signals received by sensors, is adopted as a data source. It can generate diverse ultrasonic echo signals, whether there is aliasing or not, as a training set via adjusting the parameters of the model and adding noise with different Signal-to-Noise Ratios (SNRs). According to [16], the model is expressed as

(3) $$S\left(\theta ;t\right)=u\left(t-\tau \right)\cos\left(2\pi f_{0}\left(t-\tau \right)+\phi \right)+w\left(t\right),$$

where u(t−τ) is the envelope of the echo, given by

(4) $$u\left(t-\tau \right)=\beta e^{-\alpha {\left(t-\tau \right)}^{2}},$$

$\theta =\left[\beta ,\alpha ,\tau ,f_{0},\phi \right]$ and w(t) is additive white Gaussian noise. Therefore, the data set from Equation (3) can be utilized to train and test the proposed network. Details of experiment are shown in Section 3.

According to [20], TOF is considered as the time delay between the time when the transducer emits ultrasonic signals and the time when the sensor receives the corresponding echo reflected from the casing. Therefore, to train D-net, we divide the sample points of echo signals into two classes which are marked as black part and red part, as shown in Figure 3. After being trained, the target of this net is to accomplish the sampled-pointwise classification and precisely determine the TOF in a complex environment.

To test the practical performances in the actual ultrasonic signals, we collect some echo signals acquired from an ultrasonic image logging tool. More details will be shown in Section 3.

## 3. Tests and Results

### 3.1. Experiments Description

**Figure 5:**
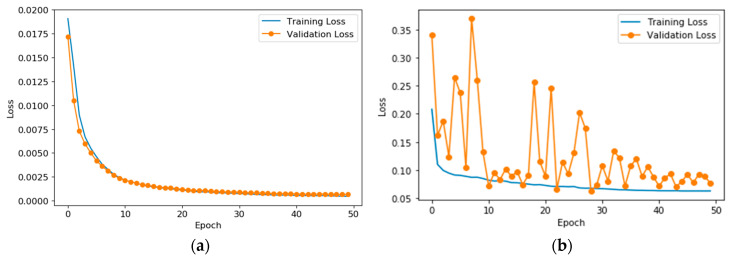
The training progress of two subnets. (**a**) S-net, (**b**) D-net.

As mentioned in Section 2.4, we utilize Equation (3) to simulate the ultrasonic echo signals reflected from the casing by composing ultrasonic signals from different linear combinations of simulated echoes. In total, 10,000 sets of data are generated, which cover almost all possible situations in the echoes, involving whether they are overlapping or not, different SNRs, normalized amplitudes, time delay, etc. In this experiment, the parameter of center frequency is selected as 1 MHz, and the sampling frequency is 4 MHz. The sampling number of a single simulated signal is 400, and its time length is 100 μs. For two subnets in WT-net, we train and validate them independently and compare them with classical methods, respectively. All data are divided into the training set, validation set and testing set, having 8000 sets, 1000 sets and 1000 sets, respectively. In the training set and validation set, according to the location of sample points corresponding to TOF, we divide them into two categories marked red label and black label, respectively, as ground truth. The training parameters of the experiment are shown in Table 3. Moreover, the training progress of two subnets is illustrated in Figure 5. 

### 3.2. Overlapping Echo Separation Test

**Figure 6:**
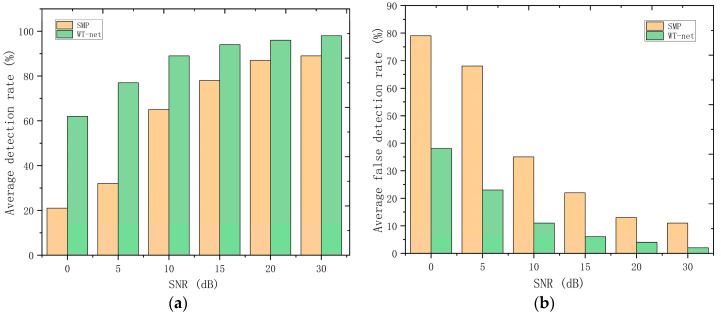
Performance of the methods with two single echo signals mixed under different SNRs: (**a**) average detection rate, (**b**) average false detection rate.

In this section, the separation performance of overlapping ultrasonic echo signals via the proposed method is evaluated and compared with the support matching pursuit (SMP) method [15]. Considering that the composing echoes of the received signal are known, the performance is computed under different SNRs in terms of detection and false discovery rates. The proposed network and the SMP method are tested in the same testing set, which has 1000 sets of echo signals data. The fraction of detections from the number of original echoes is the detection rate, and the fraction of false discoveries from the number of discoveries is the false detection rate. For simulating the actual background in the thickness measurement of pipelines, the number of original echoes is set to 2, and they are mixed with different TOF and SNRs. Figure 6 demonstrates the performance of two methods under different SNRs.

As is shown in Figure 6, contrasted with the classical method, the proposed network outperforms because the network with convolution layer can capture local features hidden in noise when it is already trained. Based on 1D-CNN, S-net is powerful enough to reveal the law of signal distribution, which is incomparable to the method based on dictionary matching.

### 3.3. TOF Determination Test

**Figure 7:**
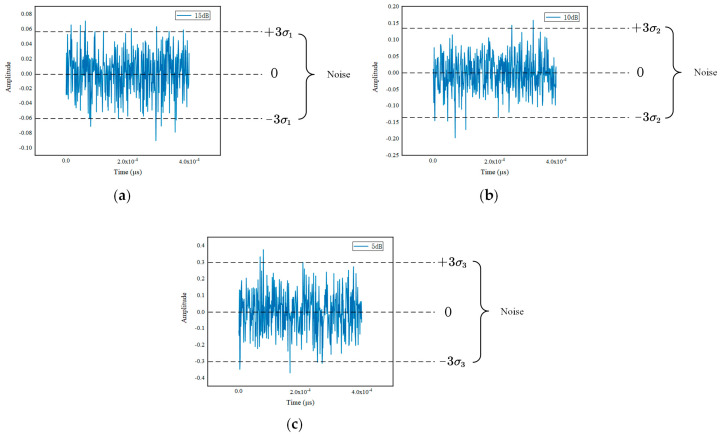
The distribution of Gaussian white noise. (**a**), (**b**) and (**c**) correspond to 15 dB, 10 dB, 5 dB, respectively.

**Figure 8:**
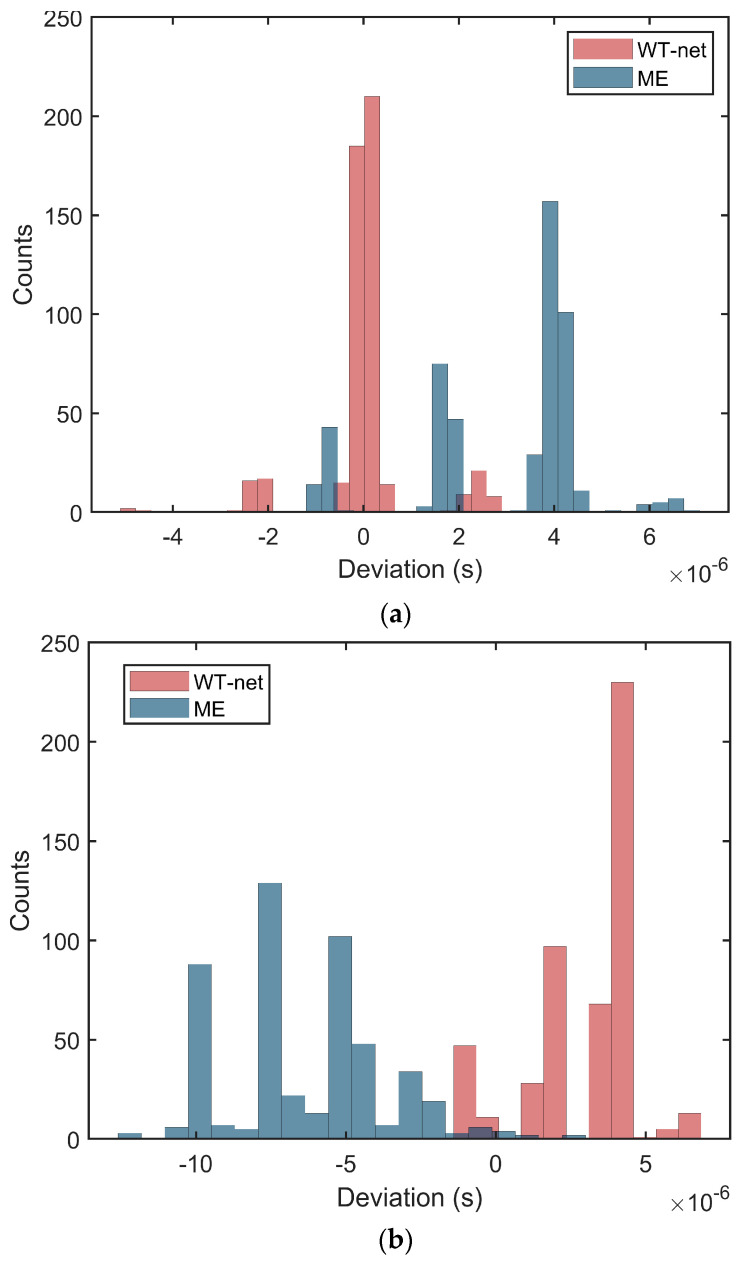

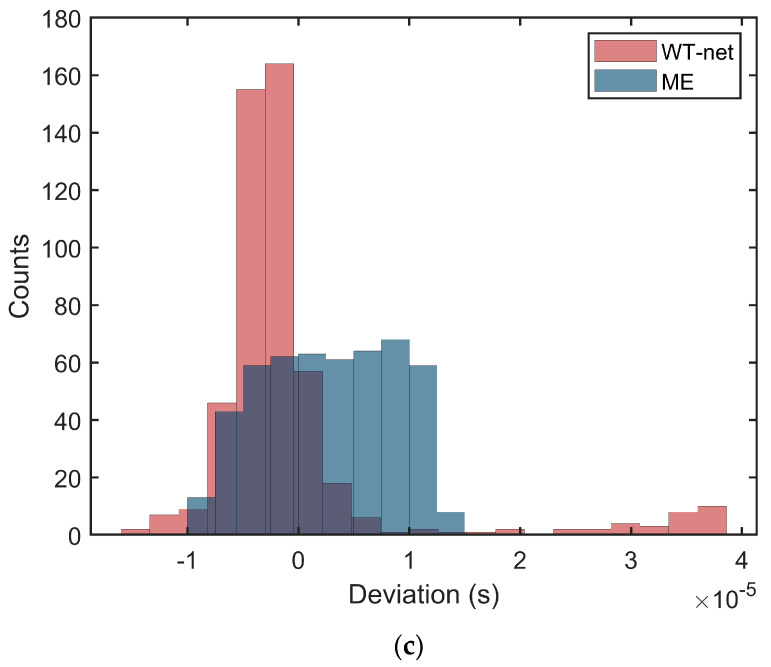
Deviations between the measured time and standard time under different SNRs by two methods: (**a**) 15 dB, (**b**) 10 dB, (**c**) 5 dB.

For assessing the performance of the D-net in TOF estimation, the separated signals from S-net are used by comparing them with the model-based estimation method (ME) [18]. A total of 500 ultrasonic echo signals are acquired in the testing set under different SNRs, which contain 15 dB, 10 dB, 5 dB for TOF estimation. These noises are used to assume that the arithmetic mean of the measurements is 0 and the variation around it is the power spectral density (PSD) of noises, as shown in Figure 7 [30]. Figure 8a–c illustrates the distribution of deviation between measured time $T_{m}$ and standard time $T_{s}$ indicated by $\Delta t=\;T_{s}-\;T_{m}$, which is obtained from the WT-net and the ME. The abscissa denotes the Δt, and the ordinate denotes the count of each Δt. As is shown in Figure 8, with the decrease in SNRs, the Δt in both methods increases, but our method performs better than the ME in measurement accuracy and anti-noise invariably. 

### 3.4. Results of Practical Tests in Pipelines

**Figure 9:**
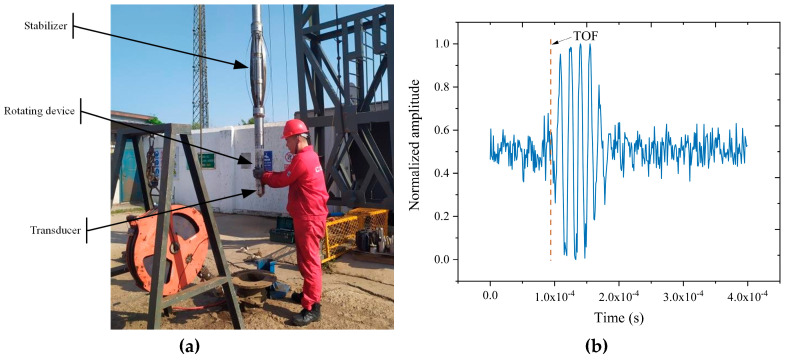
(**a**) The actual test of MUIL in the experimental well, (**b**) A typical ultrasonic echo signal collected by MUIL.

**Figure 10:**
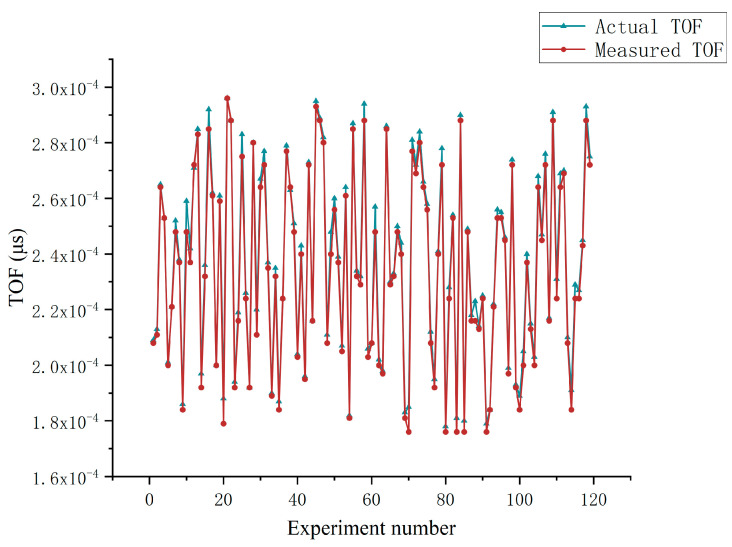
The distribution of measured TOF and actual TOF (Orange dots). Blue line denotes baseline where the measured TOF equals the corresponding actual TOF.

To analyze the actual performance of the proposed network, this section discusses the actual experiments performed for the buried pipelines. The data of ultrasonic echo signals are sampled by the Multi-functional Ultrasonic Imaging Logging Tool (MUIL) in the experimental well. The instrument is produced by China Oilfield Services Limited (COSL), also providing these actual echo signals. Figure 9a shows the actual test of the instrument in the experimental well. The dataset consists of 118 ultrasonic signals corresponding to the thickness at 118 positions in the wall, including whether overlapping signals or not. The center frequency of these signals is 1 MHz. Every signal segment lasts 100 μs. Figure 9b shows one of these ultrasonic signals. All echo signals are tested by the proposed network to calculate the deviation from the corresponding standard thickness. Figure 10 illustrates the distribution of the measured value and corresponding standard thicknesses of wall. As shown in Figure 10, The deviations are quite close between the measured TOF and the corresponding actual value, in which the deviation ranges from 1 μs to 15 μs. The mean square error (MSE) is $1.3076\times 10^{-11}\;s^{2}$.

### 3.5. Discussion of Results

According to Figure 8b,c, we find that TOFs determined by the proposed methods have fixed deviations relative to the standard values in the testing set. By analyzing the testing set, when the amplitude of echo signals is quite small, the amplitude can be submerged by noise easily. The reason for this phenomenon is that in covering all possible combinations of ultrasonic signals, we create an over-complete dataset in which there are several data without obvious features of echo signals. Accordingly, when the WT-net determines the TOFs of these echo signals, the results may deviate from the standard value but still perform better than the ME method. Furthermore, we are preparing a specific pipeline to acquire realistic ultrasonic signals and enhance dataset.

## 4. Conclusions

In this paper, a method termed WT-net, based on 1D-CNN, and semantic discrete sample points segmentation, is proposed to determine the TOF of overlapping ultrasonic echo signals in the thickness measurement of pipelines. The network consisted of two subnets, S-net and D-net, corresponding to accomplish echo separation and to TOF determination, respectively. Compared with classical methods, it can identify the ultrasonic echo signals whether overlapping or not and separate them precisely. Furthermore, differing from the model-based approach, it can determine the TOF of every single echo precisely by trainable 1D semantic segmentation, which realizes the sample pointwise classification by the definition of TOF. The results of the simulated experiment demonstrate the satisfactory performance of the proposed method.

In the next stage, in order to verify the performance in actual working environments further, the practical test will be carried out in the laboratory. We have produced a steel pipeline possessing various diameters and thickness of the wall, and the cracks in the wall are produced deliberately to test the actual performance of the proposed network. 

## Figures and Tables

**Table 1:** Specific structure of S-net.

Structure	Parameters	Remark
Input	400 × 1	Original signal
Conv_1	5 × 1 × 32	Stride: 1 × 1
LeakyReLU_1		Activation function
Maxpooling_1	2 × 1	Stride: 2 × 1
Conv_2	5 × 1 × 64	Stride: 1 × 1
LeakyReLU_2		Activation function
Maxpooling_2	2 × 1	Stride: 2 × 1
Conv_3	5 × 1 × 96	Stride: 1 × 1
LeakyReLU_3		Activation function
Maxpooling_3	2 × 1	Stride: 2 × 1
FC_1	2000 × 1	
ReLU_1		Activation function
FC_2 ^1^	800 × 1	Single separated signal

^1^ This layer is output of S-net, first half is the first component of overlapping echo, second half is another one.

**Table 2:** Specific structure of D-net.

Structure	Parameters	Remark
Input	400 × 1	Separated echo from S-net
Block1_conv1	3 × 1 × 64	Stride: 1 × 1
Block1_conv2	3 × 1 × 64	Stride: 1 × 1
Block1_Maxpooling	1 × 2	Stride: 1 × 2
Block2_conv1	3 × 1 × 128	Stride: 1 × 1
Block2_conv2	3 × 1 × 128	Stride: 1 × 1
Block2_Maxpooling	1 × 2	Stride: 1 × 2
Block3_conv1	3 × 1 × 256	Stride: 1 × 1
Block3_conv2	3 × 1 × 256	Stride: 1 × 1
Block3_conv3	3 × 1 × 256	Stride: 1 × 1
Block3_Maxpooling	1 × 2	Stride: 1 × 2
Block4_conv1	3 × 1 × 512	Stride: 1 × 1
Block4_conv2	3 × 1 × 512	Stride: 1 × 1
Block4_conv3	3 × 1 × 512	Stride: 1 × 1
Block4_Maxpooling	1 × 2	Stride: 1 × 2
Conv_5	3 × 1 × 512	Stride: 1 × 1
Upsampling	1 × 2	Stride: 1 × 2
Conv_6	3 × 1 × 256	Stride: 1 × 1
Upsamping	1 × 2	Stride: 1 × 2
Conv_7	3 × 1 × 128	Stride: 1 × 1
Upsampling	1 × 2	Stride: 1 × 2
Conv_8	3 × 1 × 64	Stride: 1 × 1
Upsamping	1 × 2	Stride: 1 × 2
Conv_9	3 × 1 × 32	Stride: 1 × 1
Conv_9	3 × 1 × 2 ^1^	Stride: 1 × 1
Softmax_1	400 × 1	Output classification of points

^1^ 2 denotes two classifications.

**Table 3:** Training parameters.

Experiments	Parameters
Operating system	Windows 10
Deep learning framework	Keras
CPU	i9 9900k
GPU	RTX 2080Ti
Training method	End-to-end
